# Kaposi's Sarcoma-Associated Herpesvirus Hijacks RNA Polymerase II To Create a Viral Transcriptional Factory

**DOI:** 10.1128/JVI.02491-16

**Published:** 2017-05-12

**Authors:** Christopher Phillip Chen, Yuanzhi Lyu, Frank Chuang, Kazushi Nakano, Chie Izumiya, Di Jin, Mel Campbell, Yoshihiro Izumiya

**Affiliations:** a Department of Dermatology, University of California Davis School of Medicine, Sacramento, California, USA; b Department of Biochemistry and Molecular Medicine, University of California Davis School of Medicine, Sacramento, California, USA; c College of Veterinary Medicine, Nanjing Agricultural University, Nanjing, People's Republic of China; d University of California Davis Comprehensive Cancer Center, Sacramento, California, USA; University of Southern California

**Keywords:** KSHV, transcription, Kaposi's sarcoma-associated herpesvirus, RNA polymerase II, reactivation, transcriptional factory, regulation of gene expression

## Abstract

Locally concentrated nuclear factors ensure efficient binding to DNA templates, facilitating RNA polymerase II recruitment and frequent reutilization of stable preinitiation complexes. We have uncovered a mechanism for effective viral transcription by focal assembly of RNA polymerase II around Kaposi's sarcoma-associated herpesvirus (KSHV) genomes in the host cell nucleus. Using immunofluorescence labeling of latent nuclear antigen (LANA) protein, together with fluorescence *in situ* RNA hybridization (RNA-FISH) of the intron region of immediate early transcripts, we visualized active transcription of viral genomes in naturally infected cells. At the single-cell level, we found that not all episomes were uniformly transcribed following reactivation stimuli. However, those episomes that were being transcribed would spontaneously aggregate to form transcriptional “factories,” which recruited a significant fraction of cellular RNA polymerase II. Focal assembly of “viral transcriptional factories” decreased the pool of cellular RNA polymerase II available for cellular gene transcription, which consequently impaired cellular gene expression globally, with the exception of selected ones. The viral transcriptional factories localized with replicating viral genomic DNAs. The observed colocalization of viral transcriptional factories with replicating viral genomic DNA suggests that KSHV assembles an “all-in-one” factory for both gene transcription and DNA replication. We propose that the assembly of RNA polymerase II around viral episomes in the nucleus may be a previously unexplored aspect of KSHV gene regulation by confiscation of a limited supply of RNA polymerase II in infected cells.

**IMPORTANCE** B cells infected with Kaposi's sarcoma-associated herpesvirus (KSHV) harbor multiple copies of the KSHV genome in the form of episomes. Three-dimensional imaging of viral gene expression in the nucleus allows us to study interactions and changes in the physical distribution of these episomes following stimulation. The results showed heterogeneity in the responses of individual KSHV episomes to stimuli within a single reactivating cell; those episomes that did respond to stimulation, aggregated within large domains that appear to function as viral transcription factories. A significant portion of cellular RNA polymerase II was trapped in these factories and served to transcribe viral genomes, which coincided with an overall decrease in cellular gene expression. Our findings uncover a strategy of KSHV gene regulation through focal assembly of KSHV episomes and a molecular mechanism of late gene expression.

## INTRODUCTION

Gene expression is regulated by the formation of active chromatin hubs (ACHs) at enhancer regions of the genome, where many tissue-specific gene promoters are brought into proximity (1). The idea of the formation of ACHs is based on the fact that the protein concentration of many nuclear factors is below the dissociation constant of protein-protein or protein-DNA interactions (2). Accordingly, it is necessary to have mechanisms to increase the local concentration of nuclear factors at a given chromatin site for continuous and effective gene transcription. Transcription factors locate their binding sites by three-dimensional scanning of nuclear space and leads to the formation of productive transcription complexes on DNA through an inherently dynamic process (3, 4). Factors that bind transiently to their recognition sites will eventually dissociate if secondary factor(s) do not also bind within a certain cycling time. Thus, the concentration of transcription enzymes and cofactors near transcription initiation sites is a very sensitive limiting factor in the efficiency of transcription (5).

Herpesviruses are known to establish replication compartments (RCs), which are nuclear structures for viral replication. RCs are the result of the assembly of cellular and viral proteins, and they function as factories to facilitate efficient viral genome replication, gene expression, and RNA export (6). Studies have also shown the involvement of RCs in the disruption of cellular pathways that are associated with antiviral defense and biosynthesis by sequestration of key components of the pathways (7, 8). RCs can be established within the nucleus or in the cytoplasm depending on the type of virus. The formation of RCs is mainly studied and characterized in DNA viruses, including the human simplex virus (HSV) and human cytomegaloviruses, as well as nucleocytoplasmic large DNA viruses. Formation of RCs by KSHV has also been studied by transient-transfection analyses, and there is evidence for at least six viral proteins involved in DNA replication that are associated with the RC (9). These include single-stranded DNA binding protein (SSB; open reading frame 6 [ORF6]), polymerase processivity factor (PPF; ORF59), DNA polymerase (Pol; ORF9), primase associated factor (PAF; ORF40/41), primase (PRI; ORF56), and helicase (HEL; ORF44) (9). All three families of herpesvirus are known to establish RCs adjacent to promyelocytic leukemia protein nuclear bodies (PML-NBs) (6). PML-NBs are known to be recruited to incoming viral genomes as an antiviral response, and the virus may take advantage of this cellular reaction for their own use (8, 10).

KSHV is the eighth member of the human herpesvirus family identified in 1994 (11). Infection by KSHV is etiologically linked to the development of Kaposi's sarcoma (KS), an angioproliferative and inflammatory lesion of the endothelium that is the most common neoplasm occurring in untreated AIDS patients (12, 13). KSHV also strongly associates with two human lymphoproliferative diseases: primary effusion lymphoma (PEL) and AIDS-related multicentric Castleman's disease (14–17). Unfortunately, both of these cancers have generally poor outcomes and short median survival times, complicated by their association with AIDS. Understanding how KSHV replicates in infected cells is thus very important to find a strategy to prevent KSHV-associated malignancies.

Like all herpesviruses, the KSHV life cycle consists of two phases, known as latency and lytic replication. In latency, the viral genome persists in the host as nuclear episomes, and its expression is largely silenced except for a few genes (12, 13). As a result, viral particles are not produced, and the latent infected cell presents only a few viral proteins. KSHV lytic replication phase is initiated by the expression of a single viral protein, K-Rta. K-Rta is both necessary and sufficient to induce lytic reactivation of the latent KSHV genome in the BCBL-1 cell line model, as well as in a *de novo* infection model (18–22). K-Rta is classified as an immediate early gene and its coding sequence is separated into two exons (21, 23). The K-Rta promoter is also activated by K-Rta itself to amplify its own expression (24). Various K-Rta responsive promoters have been identified *in vitro* and *in vivo* (21, 25–29), thus expression of K-Rta triggers a cascade of viral gene expression.

Recent genomic studies demonstrated that host cell chromosomes physically redistribute to form genomic hubs in response to external stimuli (30–35). Transcriptionally active genomic sites were marked by RNA *in situ* hybridization and the association of genomic regions within the nucleus could be examined. These studies showed that inducible gene promoters frequently translocated (in proximity) to regions of active gene transcription and formed genomic hubs (34). It remains unknown whether viral episomes are regulated in a similar manner. Because of the small size of its genome and well-established analytical tools (20, 36, 37), the KSHV episome represents an ideal system to investigate the molecular mechanisms of chromosome movements and the downstream effects on gene expression.

In this study, we developed an approach to fluorescently label transcribing KSHV episomes in infected cells. We also established a technique to visualize ongoing viral DNA replication by targeting single-stranded DNA. By imaging KSHV transcription *in situ*, we found that KSHV episomes formed “transcriptional factories” during reactivation, toward which the translocation of a significant fraction of cellular RNA polymerase II (RNA Pol II) was observed. Based upon the results of this study, we postulate that the redistribution of viral episomes in the cell nucleus and recruitment of RNA Pol II to generate viral transcriptional factories is a fundamental molecular mechanism for effective viral gene expression.

## RESULTS

### Establishment of immune-FISH to monitor reactivating episomes in primary effusion lymphoma cells.

**FIG 1:**
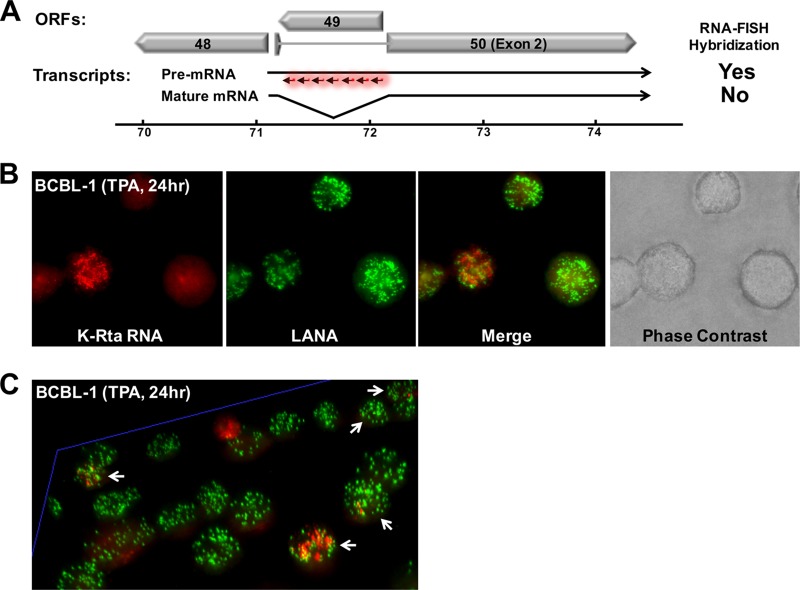
Establishment of immune-FISH approach. (A) Design of RNA-FISH probes. Fluorescence-labeled DNA oligonucleotides were designed to hybridize to the intron region of the K-Rta transcript. Premature and mature mRNA encoding K-Rta are illustrated. ORFs located around ORF50 (K-Rta) are also depicted. (B) Visualization of active episomes *in situ*. Unsynchronized BCBL-1 cells were treated with TPA (20 ng/ml) for 24 h and stained by immune-FISH. LANA was stained with specific antibody and K-Rta transcripts were visualized by fluorescent labeled oligonucleotides. A phase-contrast image is also shown. (C) Multicolor fluorescence image of BCBL-1 cells viewed in 3D perspective. Reactivation was induced by TPA (20 nM) for 24 h. White arrows indicate reactivating cells. Green, LANA; red, K-Rta RNA.

Current methods to study viral gene expression rely primarily on quantitative reverse transcription-PCR (qRT-PCR) of total RNA isolated from a mixed population of both latent infected cells and reactivating cells. Using such an approach, it is impossible to know the cell-to-cell variation in response to stimuli—much less the response of individual viral genomes (episomes) in a particular cell. Knowing how and where transcription takes place in the nucleus can provide useful insight into the regulation of viral gene expression. Are only a few episomes actively transcribing RNA, or do all the episomes in the cell transcribe viral genes simultaneously, and if so, how? To answer such questions, we applied an RNA-FISH approach to visualize viral transcripts. This technique was first described in a study of cellular gene regulation, in which target transcripts were marked near the transcribing genomic locus *in situ* by generating probes specifically to intronic regions (30, 34). Using the same approach, we designed RNA-FISH probes for the K-Rta intron region (Fig. 1A and Table 1), which allowed us to specifically tag pre-mRNAs immediately after transcription from viral DNA (but before splicing and export to the cytoplasm). Transcripts become visible only when a sufficient number of fluorescent oligonucleotide probes are hybridized to the same K-Rta intron region; this mechanism increases the specificity of hybridization signals. KSHV reactivation was induced for 24 h by incubation with 12-*O*-tetradecanoyl-phorbol-13-acetate (TPA). In combination with LANA immune staining to mark the location of viral episomes in infected cells, we successfully obtained the first images of reactivating KSHV episomes *in situ* (Fig. 1B). The results show RNA molecules as single dots. The image also reveals that not all of the episomes in a single cell react uniformly to TPA stimulation (Fig. 1C and enlarged figure is shown in Fig. S1 in the supplemental material). Three-dimensional (3D) imaging shows that some KSHV episomes (labeled in green) were adjacent to transcripts (labeled in red), suggesting that these episomes were the likely origin of the RNAs (Fig. 1C). Brighter red signals correspond to higher RNA concentration and could represent points of origin for transcriptional activity. Possibly due to RNA mobility within the nucleus, not all of the RNA molecules were expected to colocalize with LANA signals. Also, as expected, most BCBL-1 cells did not reactivate (as demonstrated by the absence of red signal) with a single TPA treatment. The results also indicated a heterogeneous KSHV reactivation, because a different number of RNA molecules (red dots in cells designated with white arrowheads, in Fig. 1C) were present in the respective cells. Finally, we also noticed that LANA dots tend to aggregate where a larger number of red punctate were present (see below).

**TABLE 1:** FISH probe sequences

Probe	Sequence (5′-3′)
KSHV Rta_1	CCCACCTACACCATTGTAAA
KSHV Rta_2	GTAGAGCTTGGCGAACTCTG
KSHV Rta_3	TTAATAAGAGCCCTGACACC
KSHV Rta_4	CTTTGGTCAAGTACACCGAA
KSHV Rta_5	TACAGACCCAACCTAGGTTC
KSHV Rta_6	GATCCTTTTTTGCCTGGTAC
KSHV Rta_7	AACTCTCCTGAAAAGCACCG
KSHV Rta_8	TTGTCCACATAATCAGCACG
KSHV Rta_9	CGGTGCATTTACGAGCAGAA
KSHV Rta_10	CAGCTGTCGTTCAGATGTAC
KSHV Rta_11	ATTTTAACGTGCAGCTGAAC
KSHV Rta_12	TACGAGGACTTTCAGGATAC
KSHV Rta_13	GATGGCCAGCGTGGTAAAAA
KSHV Rta_14	AACAACGCACAACGGGACGT
KSHV Rta_15	GCTCCGAAGTTAGGGATATC
KSHV Rta_16	AGTTTTGACTCTCCAATACC
KSHV Rta_17	AGAGCTCATTAAGGGACTGC
KSHV Rta_18	CTGGCTGCATAGGTTTTGAG
KSHV Rta_19	TATTAACGGCATGCTGCCAC
KSHV Rta_20	GCAACGACAAGATAAGGGGT
KSHV Rta_21	CTGCAGCAGTTGTACAAACT
KSHV Rta_22	TAGGGCGAATTTGGCTTATT
KSHV Rta_23	GACAAAGCGAGCCGTACCTG
KSHV Rta_24	ATTCAGATATTGGTCCAGCA
KSHV Rta_25	GACCCTATACCTTGCAAAGA
KSHV Rta_26	TACCCGCTCTAAACGTTGAG
KSHV Rta_27	TTCAGGAGTTAGATACCCTG
KSHV Rta_28	TTTAGGTATCATTACCCGTC
KSHV Rta_29	AAGATCACCTGTTCAACCAT
KSHV Rta_30	ACAATGACATCGAGAAGGCC
KSHV Rta_31	AGAGTGGCGTGTCATAGTTT
KSHV TR_1	TAAAACAGGGGGGGGGGATG
KSHV TR_2	CACGCCTACTTTTTTTTTCG
KSHV TR_3	CTGGACACTACGTGAACACC
KSHV TR_4	TCAGTGCTTGCTACGTGGAG
KSHV TR_5	CTTGTGTGTGAGCCTGTTTG
KSHV TR_6	TCTACTGTGCGAGGAGTCTG
KSHV TR_7	CCGCGGGAGAAAACGAAAGC
KSHV TR_8	CTCGCACAGTAGAGAGAGGG
KSHV TR_9	CAGGCTCACACACAAGACA
KSHV TR_10	ACGTAGCAAGCACTGAGGAG
KSHV TR_11	AAAAAGTAGGCGTGGCCTAG

### Formation of KSHV transcriptional factories.

**FIG 2:**
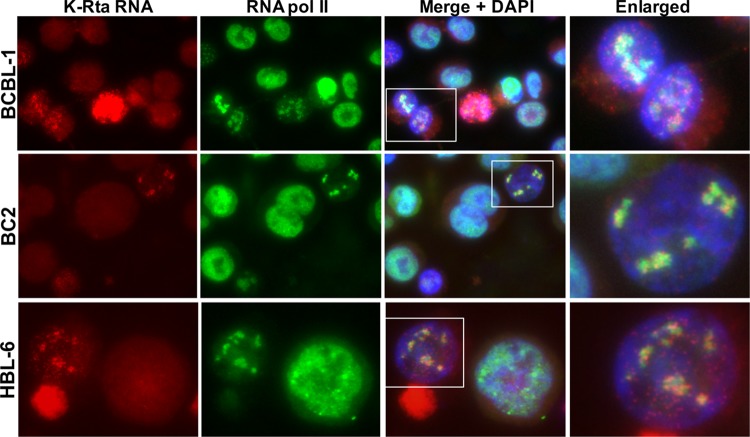
Hijacking cellular RNA Pol II. Cellular RNA Pol II (green) colocalizes with K-Rta RNA (red). The indicated PEL cells were stimulated by a mixture of TPA (20 nM) with sodium butyrate (1 mM) for 4 h and stained at 24 h after the end of stimulation. DNA was counterstained with DAPI. Areas defined by rectangles in the merge + DAPI images are enlarged at the far right.

Stimulation of cellular transcription has been shown to generate so called “transcriptional factories,” where an increased signal intensity of RNA Pol II was observed by immunofluorescence staining (34, 38). We reason that K-Rta RNA, which has immediately been transcribed from the viral genome, *should* be in close proximity with RNA Pol II. Accordingly, we stained for RNA Pol II and probed for K-Rta RNA intron-containing transcripts by RNA-FISH. The results showed colocalization of K-Rta RNA with cellular RNA Pol II in BCBL-1 cells (Fig. 2). Accumulated K-Rta RNA signals, presumably at actively transcribing sites, colocalized very well with RNA Pol II, while some distantly spaced K-Rta RNA did not. To ensure that we were not observing a cell line-specific phenomenon, we also repeated the study with two other PEL cell lines, BC2 and HBL-6. KSHV reactivation by TPA and sodium butyrate again induced formation of RNA Pol II foci, which colocalized with K-Rta RNA (Fig. 2). Linear intensity plots confirmed the overlapping distribution of RNA Pol II and K-Rta transcripts (see Fig. S2A in the supplemental material). The results also showed a significantly lower RNA Pol II signal intensity in reactivating cells compared to nonreactivating cells (Fig. 2). The number of cells that had little to no detectable RNA Pol II signal increased at later time points in postreactivation (data not shown). Translocation of cellular RNA Pol II required KSHV reactivation because latent cells (defined by absence of red dots) rarely exhibited punctate staining patterns of RNA Pol II (8.1%, Table 2), while RNA Pol II exhibited punctate staining patterns in approximately 80% of reactivating cells (Fig. 2 and Table 2). As controls, we performed the same set of experiments with slides of BJAB cells (KSHV-negative cells) and RNase-treated BCBL-1. The results showed the absence of K-Rta RNA staining, ensuring the specificity of the RNA FISH signal (see Fig. S2B and C in the supplemental material).

**TABLE 2:** Relationship between KSHV reactivation and RNA polymerase II translocation^*a*^

PEL cell type and analysis	BCBL-1/Pol II	HBL-6/Pol II	BC2/Pol II	BC3/Pol II	Total
PEL cells with K-Rta RNA signal					
K-Rta RNA (+) cells (with red dots)	142	27	143	180	492
RNA Pol II translocation	119	16	113	155	403
RNA Pol II translocation (%)	83.8	59.3	79.0	86.1	81.9
PEL cells without K-Rta RNA signal					
K-Rta RNA (–) cells (without red dots)	734	279	925	663	2601
RNA Pol II translocation	37	33	56	98	224
RNA Pol II translocation (%)	5.0	11.8	6.1	14.8	8.6

a Numeric data values are expressed as number of cells, unless noted otherwise in column 1.

**FIG 3:**
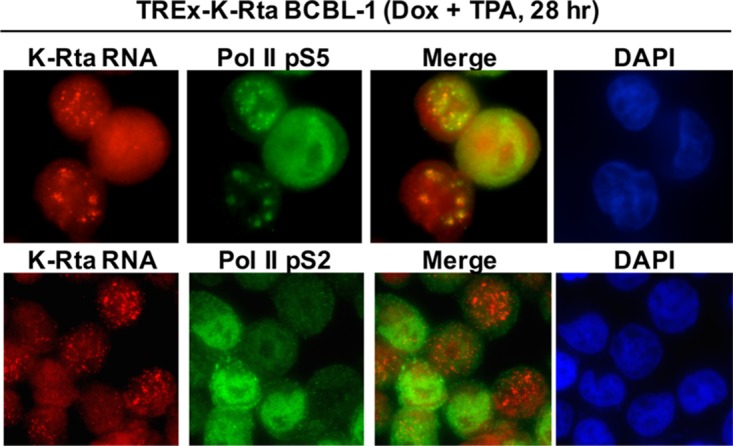
The transcriptional factory predominantly colocalizes with the RNA Pol II p-S5 form. Immune-FISH was performed with phospho-specific RNA Pol II antibodies. KSHV reactivation was induced in TREx-K-Rta BCBL-1 by combination of Dox and TPA for 4 h, and slides were prepared after 24 h after the end of the stimulation. KSHV transcribing sites were marked by RNA-FISH with K-Rta intron probes.

Next, we performed immune-FISH with RNA Pol II phospho-specific antibodies. By using the phospho-specific RNA Pol II antibodies, we examined the relationship between K-Rta RNA *in situ* signal with the RNA Pol II status, in addition to confirming the results with different antibodies targeting the same molecule. C-terminal domain (CTD) of RNA Pol II is known to be phosphorylated and position of CTD phosphorylation sites (p-S2, p-S5) regulates recruitment of different protein complexes and thus regulates the function of RNA Pol II. The results demonstrated that S5-phosphorylated, but not S2-phosphorylated RNA Pol II preferentially colocalized with K-Rta RNA. Interestingly, RNA Pol II S2 signals in proximity to K-Rta RNA signals were significantly lower (Fig. 3).

### Proteasomes degrade cellular RNA Pol II during KSHV reactivation.

**FIG 4:**
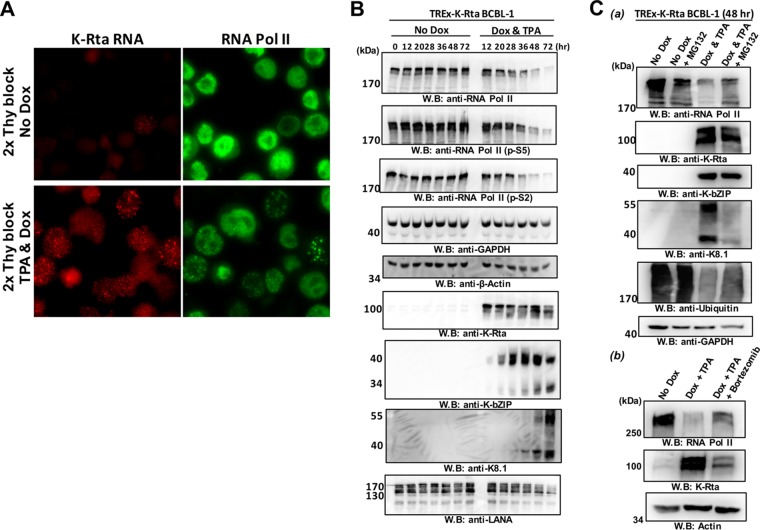
Degradation of RNA Pol II by the proteasome in KSHV-reactivating cells. (A) KSHV reactivation in synchronized TREx-K-Rta BCBL-1 after treatment with a combination of TPA and doxycycline. The cell cycle was synchronized with a double thymidine block, and KSHV reactivation was induced by a combination of Dox (1 μg/ml) and TPA (20 ng/ml). The indicated molecules were stained with immune-FISH at 28 h postinduction. (B) Immunoblotting. TREx-K-Rta BCBL-1 cells were reactivated by TPA and Dox for 4 h after cell cycle synchronization. Cell lysates were prepared at different time points after reactivation, and 50-μg portions of total cell lysates were subjected to immunoblotting. The indicated proteins were probed with specific antibodies. No Dox, no KSHV reactivated cells; Dox & TPA, reactivated by TPA and Dox for 4 h. (C) Proteasome-mediated degradation of RNA Pol II. (a) TREx-K-Rta BCBL-1 cells were reactivated by TPA and Dox for 4 h after cell cycle synchronization. At 28 h after stimulation, MG132 (final concentration, 10 μM) was added to the culture media and remained another 20 h. The indicated proteins were probed with specific antibodies. (b) Bortezomib (final concentration, 16 nM) was added to the culture media after stimulation of KSHV reactivation for 4 h and treatment lasted another 44 h. At 48 h poststimulation, the cells were harvested, and cell lysates were prepared. The indicated proteins were probed with specific antibody.

Our immune-FISH studies showed significantly lower RNA Pol II signals in reactivating cells. To examine the possibility that this was mediated by a degradation-associated pathway, we applied a proteasome inhibitor to determine whether we could inhibit the degradation of cellular RNA Pol II. As noted above, nonreactivated cells do not exhibit RNA Pol II degradation; therefore, we needed to increase the number of KSHV reactivated cells in a given tissue culture population to examine this process with a whole population by immunoblotting. Accordingly, we implemented double thymidine blocks to synchronize the cells, followed by KSHV reactivation by the induction of K-Rta expression with doxycycline (Dox) in K-Rta inducible BCBL-1 cells (TREx-K-Rta BCBL-1). TPA was also included during reactivation to further induce reactivation. With this approach, we were able to achieve nearly 100% induction of cells expression of K-Rta RNA (Fig. 4A, bottom left panel). To prevent exogenous K-Rta overexpression, the incubation time with DOX and TPA was limited to 4 h. The K-Rta inducible cassette encodes only exon sequences of K-Rta mRNA and our RNA-FISH probes were designed to hybridize to the intron region of the K-Rta transcript (Fig. 1A), thus we still could probe endogenous K-Rta RNA expression and mark the transcribing episomes in the K-Rta inducible cells (Fig. 4A). By taking time points after reactivation, we monitored cellular and viral protein expression by immunoblotting. As expected, the amount of RNA Pol II began to decrease after approximately 28 h and became nearly undetectable at the 72-h time point (Fig. 4B). Similar results were obtained with two phospho-specific RNA Pol II antibodies, suggesting that there are no differences in RNA Pol II degradation due to the status of RNA Pol II phosphorylation. Although both actin and GAPDH were expressed at significantly higher levels in the cells, both proteins also showed a slight reduction at later time points (Fig. 4B). On the other hand, robust expression of the KSHV early gene product, K-bZIP, was detected at the 28-h time point; this was consistent with the timing of RNA Pol II accumulation. Although a significant fraction of RNA Pol II was degraded by the 72-h time point, the viral late gene product, K8.1, continued to be expressed at 72 h (Fig. 4B). On the other hand, LANA expression was induced at the 12-h time point and gradually decreased thereafter (Fig. 4B). Incubation with a proteasome inhibitor, MG132, slightly recovered the amount of RNA Pol II (Fig. 4C). We added MG132 at the 28-h time point and remained in the cultures for another 20 h. Samples were thus collected at 48 h after reactivation. Incubation of doxycycline and TPA for 4 h induced early lytic gene expression (K-Rta and K-bZIP), and there were no differences by incubation with MG132; however, expression of late gene product, K8.1, was significantly inhibited by the MG132 treatment (Fig. 4C, upper panel). The recovery of RNA Pol II was also confirmed with bortezomib, a U.S. Food and Drug Administration (FDA)-approved proteasome inhibitor. For the bortezomib treatment, K-Rta was induced in the presence or absence of bortezomib at final concentration of 16 nM, which is approximately 3 times above the 50% inhibitory concentration. These results showed that RNA Pol II degradation, at least in part, was mediated by the proteasome (Fig. 4C, lower panel).

### Biased shifting of RNA Pol II for selected gene expression.

**FIG 5:**
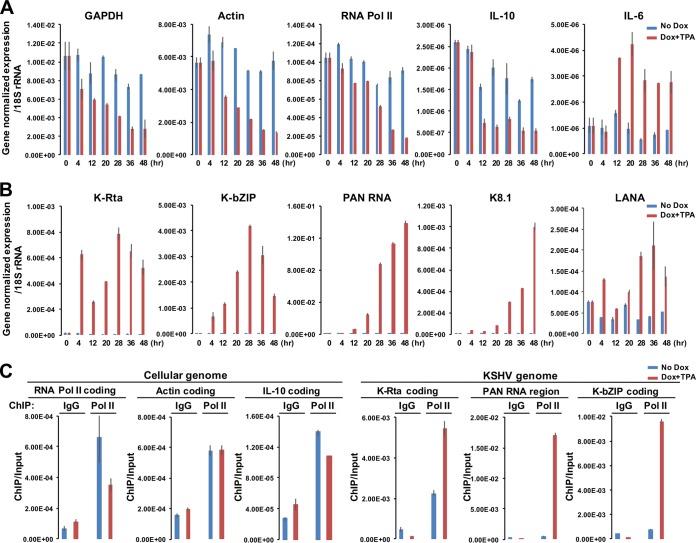
Biased shifting of RNA Pol II onto viral genomes during reactivation. (A and B) Cellular (A) and viral (B) gene expression. TREx-K-Rta BCBL-1 cells were reacted by TPA and Dox for 4 h after cell cycle synchronization. The indicated cellular or viral gene expression was measured by qRT-PCR. rRNA 18S, which is transcribed by RNA Pol I, was used for normalization. The values of gene-normalized expression at the indicated time points are shown in the bar graph. Blue, No Dox; red, Dox and TPA. (C) Chromatin immunoprecipitation. RNA Pol II occupancies at cellular or viral promoters were determined before and during reactivation (i.e., at 28 h postreactivation). ChIP assays were performed with the indicated genomic regions with specific primers. The relative enrichment over input DNA is shown.

We next examined the impact of viral transcriptional factory formation on cellular and viral gene expression. TREx-K-Rta BCBL-1 cells were reactivated by a combination of Dox and TPA for 4 h, and total RNA was extracted at different time points. Total RNA was also extracted from noninduced cells for comparison. Consistent with there being diminished levels of available RNA Pol II for cellular gene transcription, the expression of *GAPDH*, β-actin, RNA Pol II (RBP1), and interleukin-10 (IL-10) were all decreased during KSHV reactivation (Fig. 5A, red bars) compared to noninduced cells (Fig. 5A, blue bars). In sharp contrast, KSHV genomes were increasingly transcribed after stimulation (Fig. 5B, red bars). Cellular IL-6, one of the K-Rta cellular target genes (39), also showed increased gene expression with kinetics similar to that of viral early gene expression; the results indicate that not all cellular gene expression was suppressed, despite the degree and extent of RNA Pol II translocation. For these qRT-PCR studies, we used 18S rRNA as an internal control, since it is transcribed by RNA polymerase I, and we found the 18S rRNA expression to be consistent throughout the experiments. Importantly, this observed effect was not due to an artifact of using K-Rta inducible cells, because cellular small ubiquitin-like modifier (SUMO) 2 protein, which is expected to have a short half-life, exhibited drastically decreased signals in KSHV-reactivating cells but not in nonreactivated BCBL-1, when examined at the single-cell level (see Fig. S3 in the supplemental material).

Finally, chromatin immunoprecipitation was performed to examine occupancy of RNA Pol II at cellular and viral genes (Fig. 5C). The results showed that RNA Pol II occupancy on viral genomes was significantly increased during reactivation (Fig. 5C, right graphs). Compared to the cellular genome, RNA Pol II was recruited approximately 10 to 100 times more on viral genomes at 28 h postreactivation. The PAN RNA encoding region recruited the highest levels of RNA Pol II among different viral genomic regions examined (Fig. 5C), which was consistent with significantly higher copies of transcripts (Fig. 5B). Although it was not as clear as we expected from immunostaining images, RNA Pol II occupancies at cellular RNA Pol II (RBP1) and IL-10 coding region (but not actin coding region) were slightly decreased. This is partly due to the presence of nonreactivating cells in the ChIP samples. These results, in conjunction with immune-FISH images, suggest that RNA Pol II formed foci to primarily transcribe viral genomes during KSHV reactivation.

### Colocalization of LANA with RNA Pol II in reactivating cells suggests regulation of KSHV gene expression by forming active chromatin hubs with other episomes.

**FIG 6:**
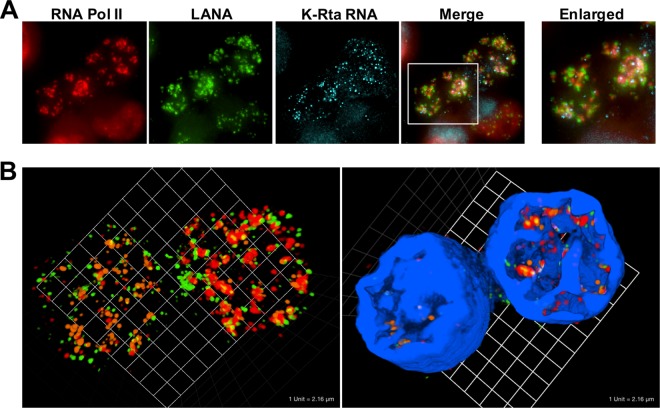
(A) RNA-FISH showing the assembly of LANA dots with RNA Pol II in BCBL-1. RNA Pol II (red immunofluorescence), LANA (green immunofluorescence), and K-Rta RNA (light blue, RNA-FISH) were visualized. BCBL-1 was stimulated with TPA and sodium butyrate for 4 h. Cells were fixed after 24 h after the end of stimulation. Merge and enlarged merge images overlaid with K-Rta RNA staining are shown. (B) 3D view of KSHV transcriptional factory. Z-stack images were taken, and 3D images without or with DAPI staining were constructed. Green, LANA; red, RNA Pol II; orange, K-Rta transcripts; blue, DAPI.

It is known that activated cellular promoters are aggregated at specific enhancer regions to form active chromatin hubs (31–33). The generation of active chromatin hubs facilitates recycling of RNA Pol II, allowing the enzyme to transcribe from assembled tissue specific or transcriptional factor bound promoters without the requirement for distant travel. The observed accumulation of RNA Pol II prompted us to examine if viral episomes are coming together to prepare active chromatin. To do this, we stained both LANA and cellular RNA Pol II along with K-Rta RNA to visualize active transcription sites. K-Rta RNA identifies the location of actively transcribing episomes, while LANA was used to mark the location of viral episomes. In previous work, we found that LANA was dissociated from the unique region of the KSHV genome; however LANA continued to bind to the TR region and remained enriched over 40-fold at the TR region during reactivation (40, 41). The immune-FISH staining clearly demonstrated that LANA dots in reactivating cells were assembling, and RNA Pol II localized very closely to the congregated LANA dots in reactivating cells (Fig. 6A). Similar to Fig. 2, RNA Pol II and K-Rta RNA dots were significantly overlapped, as expected (Fig. 6B [orange dots, K-Rta RNA; red dots, RNA Pol II; green dots, LANA]). Interestingly, the KSHV transcriptional factory appeared to occupy a multilobular space that resembles an “ant's nest” and was largely devoid of DAPI staining (Fig. 2A, and Fig. 6B, right panel with DAPI [4′,6′-diamidino-2-phenylindole]). The size and shape of this nuclear space was different in each reactivating cell. 3D fluorescence deconvolution microscopy further showed that LANA (green) and RNA Pol II (red) frequently formed ring-like shapes, and K-Rta RNAs (light blue) were often seen within the ring structure. LANA was located outside RNA Pol II to form the ring shapes, which is like a cage to trap RNA Pol II (see Fig. S4 in the supplemental material). Taken together, our results strongly suggest that actively transcribing episomes were assembled to create a viral transcriptional factory. Focal concentration of RNA Pol II near the viral episomes then facilitates viral gene expression.

### KSHV DNA replication and transcriptional factory formation.

**FIG 7:**
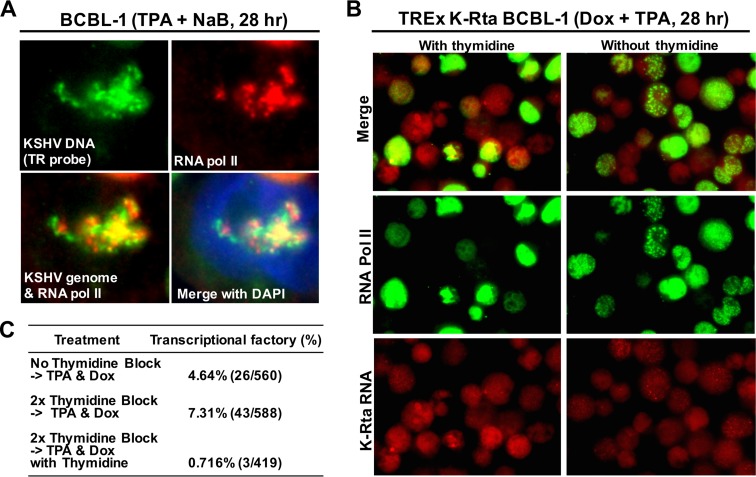
DNA replication and transcriptional factory formation. (A) Colocalization between single-stranded viral DNA and RNA Pol II. The indicated molecules were probed with either antibody or fluorescent oligonucleotide probes. Merged images: green, replicating KSHV genomes; red, RNA Pol II; blue, DAPI. (B) Transcriptional factory formation in presence or absence of thymidine. TREx-K-Rta BCBL-1 cells were reactivated in the presence or absence of thymidine. The aggregation of RNA Pol II is shown. (C) Pictures of five randomly selected fields were taken, and cells with complete colocalization of all three factors (RNA Pol II, LANA, and K-Rta RNA similar to Fig. 6) were counted as transcriptional factories. The numerical results are summarized in panel C.

Next, we examined if viral transcriptional factories are associated with KSHV DNA replication, because both biochemical reactions use the same KSHV genomes as templates. We probed ongoing KSHV DNA replication using a modified DNA-FISH approach. Fluorescence probes were generated to hybridize to the terminal repeat sequence. Targeting terminal repeat sequences increases fluorescence signals due to the presence of multiple tandem copies in a genome. Actively replicating DNA should possess single-stranded DNA (ssDNA), which would then be hybridized by the fluorescence-labeled DNA oligonucleotides without having to perform DNA denaturation. Avoiding denaturation also permitted us to perform multiplex analysis with protein staining, as well as RNA-FISH. Control studies with RNase A treatment did not eliminate the signal with the TR probes, thereby indicating the probes were hybridized to ssDNA (see Fig. S5 in the supplemental material). In fact, staining patterns were significantly different from RNA-FISH staining (see Fig. S5 in the supplemental material). DNA-FISH with RNA Pol II staining showed that RNA Pol II was recruited to the sites of KSHV DNA replication, and colocalized with single-stranded viral DNAs (Fig. 7A). Active DNA replication might be important for the assembly of RNA Pol II foci, because continuous incubation with thymidine inhibited formation of transcriptional factories ∼10-fold (Fig. 7B and C).

## DISCUSSION

It is known that gene expression is a very complex process that involves multiple biochemical reactions and physical processes, which ultimately leads to significant variability in the abundance of gene products. Collective evidence from single-cell sequencing further highlights the dynamics of transcription (42). In this study, we showed that viral episomes with identical genomic sequence in a single cell are not uniformly transcribed: some episomes appeared to produce K-Rta RNA, while others remained silent (Fig. 1C). We also observed a small number of K-Rta RNA transcripts in latent cells (Fig. 1C); these results suggest that K-Rta expression does not always lead to the initiation of viral replication, and KSHV reactivation may require a threshold amount of K-Rta protein in order to trigger the biochemical reaction to proceed.

KSHV heterogeneity of response to stimulation has also been documented by Darst et al. (43). They used MAPit single molecule foot-printing approach in conjunction with sequencing to analyze the accessibility of *M.CviPI* DNA methyltransferase. By assessing the accessibility of the enzyme to KSHV episomes, they examined chromatin structure changes in response to TPA (43). Their results showed that only ∼10% of viral episomes can transcribe following a given stimuli. The results also indicated that an open chromatin structure is a prerequisite in order for episomes to respond to an inducer of lytic transcription, although the study does not distinguish whether all episomes in the 10% of cell population possessed open chromatin structure versus whether 10% of the episomes within a cell can be accessed by RNA Pol II. Our studies agreed with these results and showed significant heterogeneity in the response within a cell, at least in the early stage of reactivation (Fig. 1C).

In our experiments, we controlled two possible biological variables within the cell population by synchronizing the cell cycle with thymidine (i.e., S phase) and by using K-Rta inducible cells to further eliminate the variation of effects on the endogenous K-Rta promoter activation. By taking these measures, we were able to achieve nearly 100% uniformity in the activation of endogenous K-Rta expression based on the signals of RNA-FISH in TREx-BCBL-1 K-Rta cells (Fig. 4A). However, we noticed that the translocation of RNA Pol II did not happen in all of the endogenous K-Rta-expressing cells. Although stimulation of reactivation by the combined treatment with TPA and sodium butyrate induced the translocation of cellular RNA Pol II in 80% of K-Rta RNA-expressing cells, the total numbers of K-Rta RNA-positive cells were actually much lower (Fig. 2, ∼20% of the total cell population). This observation suggests that an excess amount of K-Rta protein may inhibit the formation of such structures or that exogenous K-Rta expression might compromise our imaging experiments by generating multiple transcriptional foci, which are too small for us to recognize as viral transcriptional factories.

Given the observed translocation of RNA Pol II to viral genomes, we were interested in studying its impact on cellular gene transcription. As expected, most cellular gene expression, including RNA Pol II (RBP1) itself, was significantly decreased, relative to viral gene expression, during KSHV reactivation. It is clear that the formation of viral transcriptional factories and the activity of lytic viral proteins (44–47) are mechanisms cleverly designed to enhance viral transcript in infected cells. This finding also raises another interesting question as to how RNA Pol II-depleted cells can react and communicate with immune cells. We speculate that, in addition to the action of the lytic viral proteins (48), the depletion/sequestration of RNA Pol II may also contribute to inhibit cellular responses against replicating KSHV by suppressing physiological activity in the host cells.

Interestingly, the expression of some cellular genes, such as cellular IL-6, is induced during KSHV reactivation (Fig. 5A). Cellular IL-6 was previously identified as a K-Rta direct target gene and also escaped from degradation by KSHV ORF37 protein (39, 49). Considering that KSHV captured cellular IL-6 gene into its genome, the cellular *IL-6* locus might also be translocated to be neighboring with viral genomes within the transcriptional factory during KSHV reactivation. Physically neighboring cellular IL-6 gene with KSHV genome might then result in genomic recombination between host and viral genomes. A similar scenario has been suggested for cellular genes between *MYC* and *IGH* (34). These genomic regions came into close proximity when gene expression was stimulated and increased the chance for recombination (34). It will be important to study which cellular genes were selected by the virus to join their transcriptional factories. Identifying such cellular genomic regions may also provide us an insight into the molecular mechanism(s) of the factory formation. It is important to note that the observation of accumulation of cellular RNA Pol II to viral genomes is also supported by previous studies, in which viral microRNA becomes predominantly associated with the RNA-induced silencing complex (RISC) during KSHV reactivation (50). If the majority of the newly transcribed RNA is derived from viral genomic DNAs, then a large fraction of the RNAs associated with cellular RNA interacting proteins in a reactivating cell would be of viral origin.

It is known that activation of NF-κB translocates NF-κB target promoters to be in proximity to the enhancer region, where highly active RNA Pol II resides (33). Physical movement of one active promoter is important for the activation of another, and active promoters were often aggregated next to each other to form active chromatin hubs (31–35). Similarly, in HSV-1 *de novo*-infected cells, it has been shown that active transcription of the HSV-1 genome is required to form HSV-1 replication compartments (51). It remains unclear how active promoters are moved to meet each other and which genomic elements or which nuclear factors are required for the regulation. Our studies demonstrated that KSHV episomes were activated in a similar fashion, which provides us an opportunity to answer such fundamental genetic questions by using KSHV reactivation as a unique model system. The combination of a high copy number of viral genomes in a cell, easy readout of viral gene expression by qRT-PCR array, defined viral transcriptional factor necessary for triggering gene expression, and a cloned KSHV genome in a bacterial artificial chromosome to generate mutant episomes (i.e., single nucleotide variants, deletions, etc.) should provide significant advantages for these studies.

Bortezomib, a proteasome inhibitor, is an FDA-approved anti-cancer drug, which has been used to treat lymphoma patients in clinical practice (52, 53). Preclinical mouse studies for PEL showed that administration of bortezomib prolongs survival in a mouse xenograft model (54–57). We found that bortezomib partially prevented RNA Pol II degradation during KSHV reactivation (Fig. 4C); however, bortezomib treatment reduced the production of DNase I-resistant KSHV virions in the culture media by ∼5-fold at 96 h postreactivation (data not shown). We also found that KSHV, in the presence of proteasome inhibitors, failed to form clear transcriptional factories and reduced K8.1 expression (Fig. 4C and see Fig. S6 in the supplemental material). This suggests that degradation of RNA Pol II after onset of viral gene transcription may be important for DNA replication and/or effective DNA encapsidation to produce viral progeny. In addition, like the generation of DNA repair foci (58, 59), protein ubiquitinylation pathways may be important for the assembly of transcriptional factories/replication compartments and hence late gene expression. Further studies are needed to answer these questions. Nonetheless, bortezomib to target the ubiquitinylation/proteasome pathway may have potential benefits as a treatment option for PEL in clinical practice.

Phosphorylation of RNA Pol II at the CTD is associated with the functional status of RNA Pol II, namely, preinitiation complex and elongation in yeast (60). Recent studies from Nojima et al. demonstrated that yeast RNA Pol II and human RNA Pol II are regulated differently (61). The group elegantly demonstrated that RNA Pol II p-S5 is significantly enriched at exon junctions, while p-S2 is predominantly associated with polyadenylation machinery (61). Our immune staining showed that RNA Pol II p-S5 form but not p-S2 form was enriched at transcriptional factories (Fig. 3). Although we tried to confirm the observation by ChIP analyses with phospho-specific RNA Pol II antibodies, we could not differentiate the degree of RNA Pol II subtype recruitment on the KSHV genome. Our results showed that occupancies of both pS2 and pS5 forms were increased significantly during reactivation (data not shown). Another explanation of p-S5 colocalization would be that there may be a responsible kinase such as ORF36 in the viral transcriptional factory (62), which may associate with phosphorylation of RNA Pol II at the serine 5 either directly or indirectly. Given that promoter-proximal splice sites and the process of splicing can enhance transcription significantly (63, 64), it is important to examine how the virus manipulates the cellular transcriptional machinery and what is the meaning of colocalization with p-S5 signals at viral transcriptional factories.

The most important question is how such a significant fraction of cellular RNA Pol II is trapped on the KSHV genome. The trapping of RNA Pol II onto the viral genome is likely associated with the extremely high copy number of viral noncoding RNA, PAN RNA, expression (Fig. 5B) (65). Related to this, a cellular, nuclear noncoding RNA located upstream of the *MYC* gene, has been shown to play an important role in *MYC* expression (66). In that study, the authors demonstrated that the physical interaction between *MYC* and noncoding RNA expression is important for the *MYC* expression (66). We speculate that one of function of PAN RNA may be to trap cellular RNA Pol II on the KSHV genome, through DNA-RNA, RNA-protein interactions, and/or process of transcription itself (67, 68), forcing RNA Pol II to transcribe viral nuclear noncoding RNA over and over. In accord with this, our ChIP studies showed that significantly higher occupancies (>30-fold) of RNA Pol II at the PAN RNA locus (Fig. 5C). We think that there might be a reason for PAN RNA to be a noncoding nuclear RNA. Being a noncoding nuclear RNA, PAN RNA would not compete with other viral coding RNA for RNA export and translational machinery. We and others have shown that PAN RNA physically interacts with the PAN RNA coding and promoter region of the KSHV genome with chromatin isolation by RNA purification (CHIRP)-seq analyses and that K-Rta protein also directly binds to PAN RNA (69; M. Campbell et al., unpublished data). According to this hypothesis, the PAN RNA transcribing region should function as an enhancer for many of KSHV genes during lytic replication as it maintains RNA Pol II in close proximity to KSHV genomes.

In summary, we established an immune-FISH approach to study KSHV gene expression at the single-episome level. We propose that the physical accumulation of RNA Pol II near viral genomes in the infected cell nucleus represents an additional layer of KSHV gene regulation, which facilitates the reutilization and repurposing of limited quantities of cellular RNA Pol II. Identifying this molecular mechanism of viral transcriptional factory formation should lead to new strategies to inhibit herpesvirus replication.

## MATERIALS AND METHODS

### Cell culture.

BCBL-1 and BJAB cells were generous gifts from D. Ganem (University of California, San Francisco). BC2 and HBL-6 cells were obtained from Masahiro Fujimuro (Kyoto Pharmaceutical University). TREx-(F3H3)-K-Rta BCBL-1 cells that expresses Flag ×3 and hemagglutinin (HA) ×3 tags at the N-terminal region of K-Rta were generated by using TREx-BCBL-1 cells (a gift from J. Jung, University of Southern California) with Flp-recombination according to the manufacturer's protocol (Invitrogen), and cultured in complete RPMI 1640 containing 50 μg/ml blasticidin and 100 μg/ml hygromycin B. These B cell lines were grown at 37°C in the presence of 5% CO_2_ in RPMI 1640 medium supplemented with 15% fetal calf serum, 2 mM l-glutamine, and antibiotics. K-Rta expression and KSHV reactivation was induced in TREx K-Rta BCBL-1 cells with 20 ng/ml 12-*O*-tetradecanoyl-phorbol-13-acetate (TPA) and 100 ng/ml doxycycline. For other PEL cell lines—BCBL-1, BC2, and HBL-6—KSHV reactivation was induced with 20 ng/ml TPA, 1 mM sodium butyrate, or both agents in combination.

### Double thymidine block.

When applicable, a double thymidine block was used to synchronize the cell cycle and to synchronize the initiation of reactivation and increase efficacy of viral reactivation (70). TREx K-Rta BCBL-1 and BCBL-1 cells at 30% confluence were incubated with 2 mM thymidine (Sigma-Aldrich) for 18 h. The cells were washed with fresh medium to remove the thymidine and allowed to grow for 9 h. The cells were again treated with 2 mM thymidine for 18 h. After the second thymidine treatment, the cells were washed with medium to remove thymidine, and KSHV reactivation was induced for 4 h with various stimuli described in the figure legend.

### RT-qPCR.

Total RNA was purified with the RNeasy Plus minikit (Qiagen). Eluted RNA was converted to cDNA by using Superscript II reverse transcriptase (Thermo Fisher Scientific) and diluted 1:100. Portions (4 μl) of template were used for every 20-μl reaction, and all samples were analyzed in triplicate using SYBR green Supermix (Bio-Rad) on a QuantStudio 3 real-time PCR system using specific primers designed previously by Fakhari and Dittmer (37). Melting curve analysis was then performed to verify product specificity. KSHV gene expression was normalized to the cellular 18S rRNA.

### Immunoblotting.

Cells were rinsed in phosphate-buffered saline (PBS) and lysed in radioimmunoprecipitation assay buffer (25 mM Tris-HCl [pH 7.6], 1.0% NP-40, 1% sodium deoxycholate, 150 mM NaCl, 1 mM EDTA, 1 mM dithiothreitol, 0.1% sodium dodecyl sulfate [SDS]). After centrifugation (15,000 × *g* for 10 min at 4°C), the protein concentration was measured by using a Pierce bicinchoninic acid protein assay kit (Thermo Fisher Scientific), and equal amounts of total cell lysates were subjected to an SDS-PAGE (6% for Pol II and 10% for the other proteins) and subsequently transferred to a Immobilon-P polyvinylidene difluoride (EMD Millipore) using a semidry transfer apparatus (Bio-Rad). Membranes were blocked for 10 min at room temperature in TBST (20 mM Tris-HCl [pH 7.5], 137 mM NaCl, 0.05% Tween 20)–5% skim milk, followed by incubation with primary antibodies overnight at 4°C. The membranes were subsequently washed with TBST three times for 10 min each time at room temperature and then incubated with horseradish peroxidase-conjugated antibodies for 1 h at room temperature. The membranes were washed three times with TBST and visualized with enhanced with SuperSignal substrate (Thermo Fisher Scientific). Where indicated, the cells were treated with 10 μM MG132 (Sigma) or 16 nM bortezomib to inhibit proteasome. The following antibodies were used for immunoblotting: anti-K-Rta (1:5,000 [62]), anti-K8.1 (1 μg/ml; Santa Cruz), anti-K8α (K-bZIP) (1 μg/ml; Santa Cruz), anti-LANA (1:1,000; Advanced Biotechnologies, Inc.), anti-RNA polymerase II (1 μg/ml; EMD Millipore), anti-GAPDH (0.2 μg/ml; Santa Cruz), anti-β-actin (1 μg/ml; Sigma-Aldrich), anti-RNA polymerase phospho-S2 polyclonal rabbit IgG (Abcam), and anti-RNA polymerase II phospho-S5 (Abcam).

### Immuno-FISH.

At 28 h (unless otherwise stated in the legends) after the induction of KSHV reactivation, the cells were harvested and washed three times with diethylpyrocarbonate (DEPC)-treated PBS. The cells were then spotted onto coverslips and subsequently fixed with 4% formaldehyde in DEPC PBS solution for 10 min. After washing three times with DEPC-PBS, the cells were quenched with 100 mM glycine DEPC-PBS solution for 10 min. After a rinsing with DEPC-PBS, the cells were permeabilized with a 1:1 methanol-acetone solution for 15 min and washed with DEPC-PBS. Primary antibodies (anti-RNA polymerase II mouse monoclonal antibody [1:250; EMD Millipore], anti-RNA polymerase phospho-S2 polyclonal rabbit IgG [1:250; Abcam], anti-RNA polymerase II phospho-S5 [1:250; Abcam], anti-LANA rat monoclonal antibody [1:80; Advanced Biotechnologies, Inc.], and anti-Small ubiquitin-like modifier SUMO-2/3 [1:200; Thermo Fisher]) were diluted in dilution buffer (DEPC-PBS, 100 μg/ml yeast tRNA [Sigma-Aldrich]), followed by incubation for 1 h at 37°C. The slides were washed three times with DEPC-PBS and then washed three times with FISH wash buffer (2× SCC [1× SSC is 0.15 M NaCl plus 0.015 M sodium citrate], 10% formamide in DEPC–double-distilled water [dH_2_O]). Coverslips were incubated with a mixture of FISH probes and secondary antibodies in hybridization solution (10% dextran sulfate, 10% formamide, 2× SSC, 100 μg/ml yeast tRNA, 125 nM FISH probe [LGH Science, Inc.], and secondary antibodies) for 18 h at 37°C. The FISH probe sequences are listed in Table 1. The listed Quasar 570-labeled oligonucleotides were combined and used for RNA FISH studies. The slides were then washed with FISH wash buffer three times for 5 min and twice with 2× SSC. DAPI was added in 2× SSC for 5 min at room temperature to stain DNA. For DNA-FISH, probes were designed to hybridize terminal repeat sequence, and RNase A was incubated where indicated as a control. The slides were rinsed with SSC and mounted onto glass slides by using SlowFade Gold antifade reagent (Life Technologies). Fluorescence images were taken in two dimensions using a Keyence BZ-X710 or in three dimensions as follows.

### 3D fluorescence microscopy and digital image analysis.

Prepared cell specimens were imaged on a computer-automated widefield fluorescence deconvolution microscope (Deltavision personalDV; Applied Precision/GE Healthcare) equipped with a ×60, 1.42NA oil immersion objective lens, xenon arc lamp, and standard filters for DAPI (blue), FITC (green), and TRITC (tetramethyl rhodamine isothiocyanate; red-orange) channels. 3D image stacks were acquired with 0.2-μm spacing in the z-axis; and image deconvolution was performed postacquisition using on-board software (Resolve3D; Applied Precision/GE Healthcare). Image data were transferred to a separate computer for 3D image rendering and subsequent analysis using VoloCITY 3D imaging suite (Improvision; Perkin-Elmer).

### ChIP assay.

Chromatin immunoprecipitation (ChIP) assays were performed as described previously (29). The antibodies used were mouse monoclonal anti-RNA polymerase II (Upstate) or control mouse IgG (Santa Cruz). Immunoprecipitated chromatin DNA was analyzed by SYBR green-based quantitative PCR (Bio-Rad) with the primers listed in Table S1 in the supplemental material.

## Supplementary Material

Supplemental material
