# Supplementary material for: Kaposi's Sarcoma-Associated Herpesvirus Hijacks RNA Polymerase II To Create a Viral Transcriptional Factory
Source: J Virol. 2017 May 12;91(11):e02491-16. doi: 10.1128/JVI.02491-16 (PMC5432858; doi:10.1128/JVI.02491-16)
Supplement: Supplemental material [file supp_91_11_e02491-16__index.html]

Kaposi's Sarcoma-Associated Herpesvirus Hijacks RNA Polymerase II To Create a Viral Transcriptional Factory — Supplemental material 

# Kaposi's Sarcoma-Associated Herpesvirus Hijacks RNA Polymerase II To Create a Viral Transcriptional Factory

## Supplemental material

- Supplemental file 1 -

  Table S1 (Primers used for real-time PCR.)

  Fig. S1 (Adjacent localization of LANA and K-Rta RNA.)

  Fig. S2 (Linear intensity plot.)

  Fig. S3 (Effects of RNA polymerase II translocation on SUMO.)

  Fig. S4 (Higher-magnification view of KSHV transcriptional factories.)

  Fig. S5 (DNA-FISH.)

  Fig. S6 (Inhibition of transcriptional factory formation by proteasome inhibitor.)

  PDF, 4.9M
