# Supplementary material for: Kaposi's Sarcoma-Associated Herpesvirus Hijacks RNA Polymerase II To Create a Viral Transcriptional Factory
Source: J Virol. 2017 May 12;91(11):e02491-16. doi: 10.1128/JVI.02491-16 (PMC5432858; doi:10.1128/JVI.02491-16)
Supplement: Supplemental material [file JVI.02491-16_zjv999182629s1.pdf]

Supplemental 1 Table . Primers used for real-time PCR

Primer name and orientation	Sequence (5' -> 3')
K-Rta promoter, F	AGCCAGCGTATGCTTCAGG
K-Rta promoter, R	TGCCTGGACAGTATTCTCACA
PAN RNA promoter, F	GGTGGCTAACCTGTCCAAAA
PAN RNA promoter, R	CAGCGAGCACAAAATCCATA
Actin, F	AGAAGTCGCAGGACCACACT
Actin, R	GTAGAGCCCACCTTCCTTCC
RNA pol II, F	CGCTGTGTCTGCTTCTTCTG
RNA pol II, R	ACCCTCGCATATGTTTTTGC
IL-6, F	CACACAGACAGCCACTCACC
IL-6, R	TTTTCTGCCAGTGCCTCTTT
IL-10, F	TGGTGAAACCCCGTCTCTAC
IL-10, R	TTCCATCTCCTGGGTTCAAG
GAPDH, F	TCGCTCTCTGCTCCTCCTGTTC
GAPDH, R	CGCCCAATACGACCAAATCC

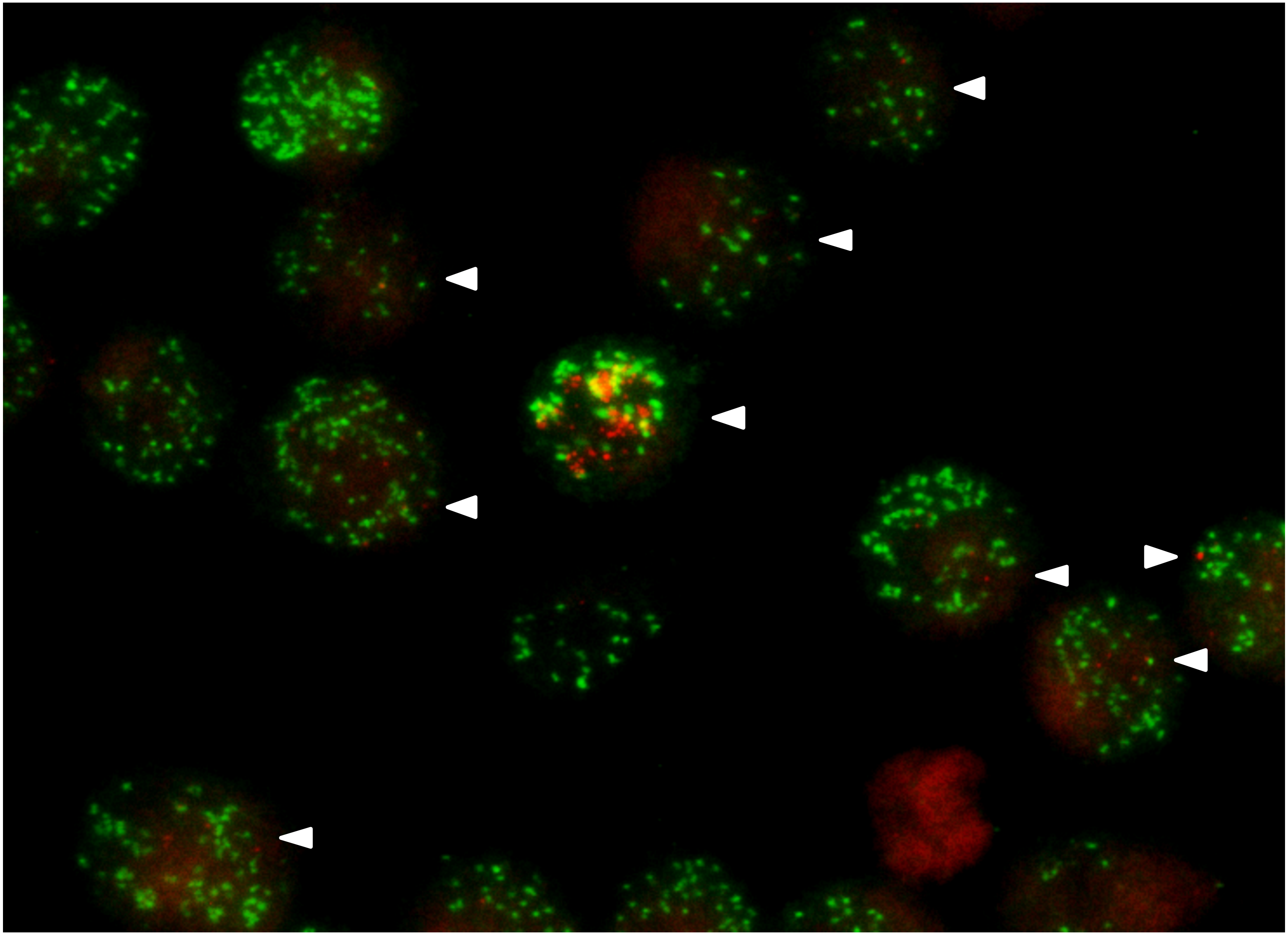

S1 Figure: Adjacent localization of LANA and K-Rta RNA. Picture presented in Figure 1 was enlarged for clearer view of adjacent localization. Immune-FISH was performed by probing K-Rta RNA (Red) and immune-staining of LANA protein (Green). Not all of episomes in a PEL cell are transcribing K-Rta RNA. BCBL-1 cells expressing K-Rta RNA is marked in white arrows.

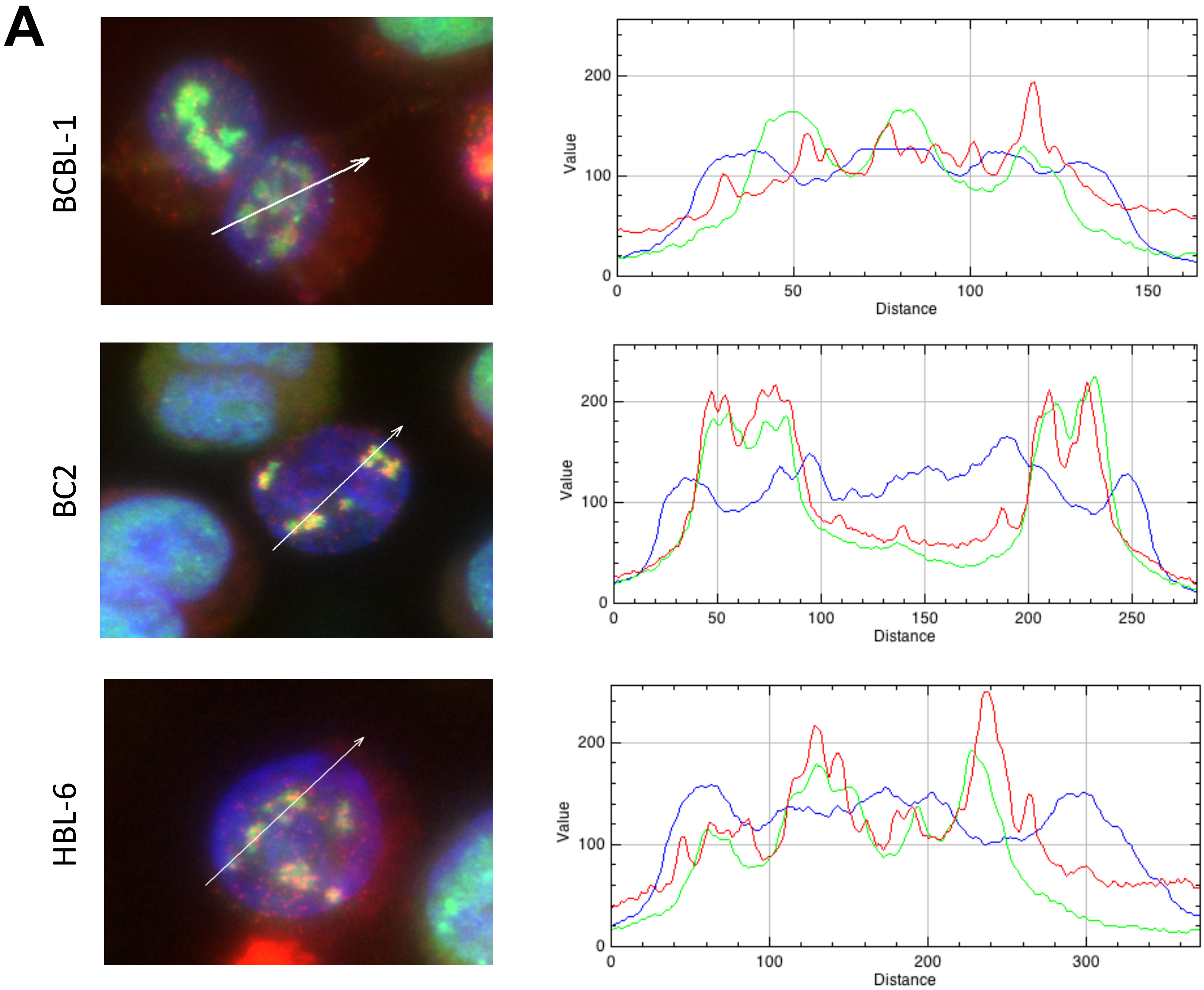
A
BCBL-1
BC2
HBL-6
Value
Distance
0
50
100
150
200
250
300

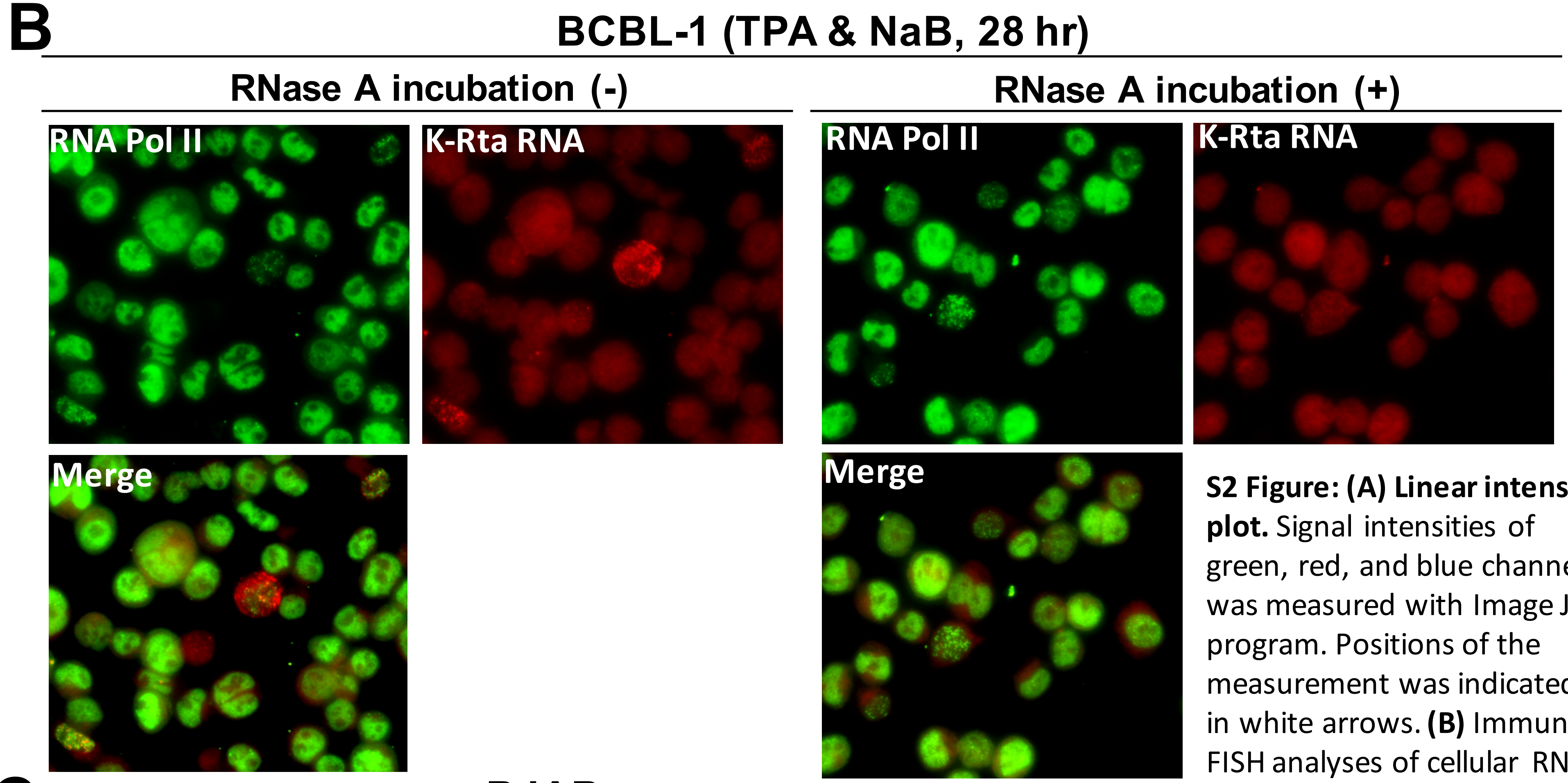

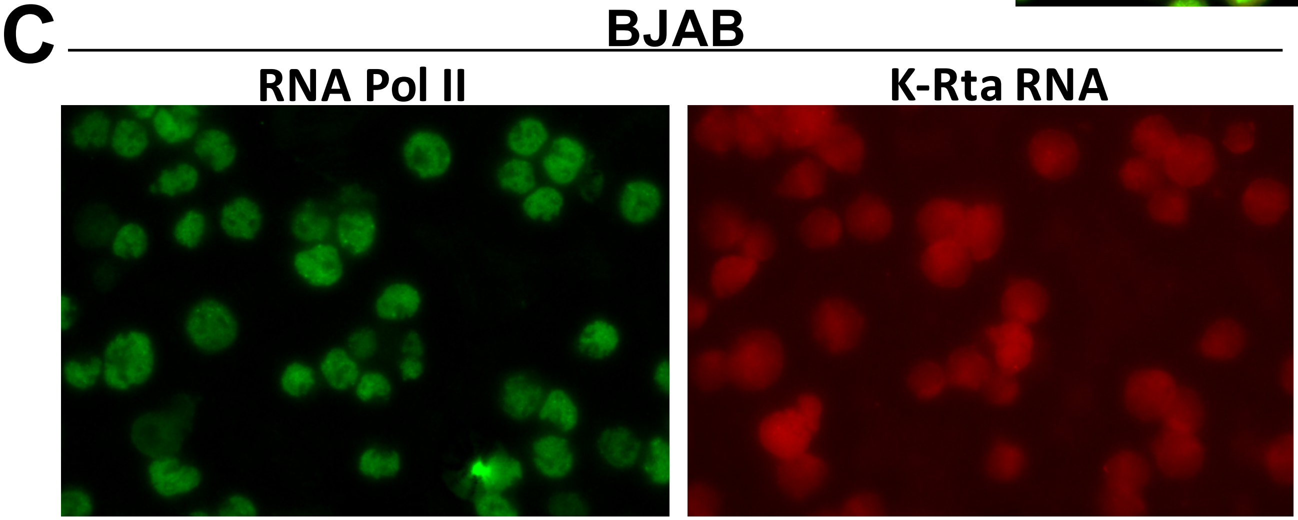

S2 Figure: (A) Linear intensity plot. Signal intensities of green, red, and blue channel was measured with Image J program. Positions of the measurement was indicated in white arrows. **(B)** Immune-FISH analyses of cellular RNA polymerase II and K-Rta RNA with BCBL-1. RNase A treatment diminished K-Rta RNA signals, which were expected to see where RNA polymerase II formed “dot-like” structures. **(C)** KSHV negative BJAB cells did not show K-Rta RNA signals. Green: RNA polymerase II, Red: K-Rta RNA FISH signals.

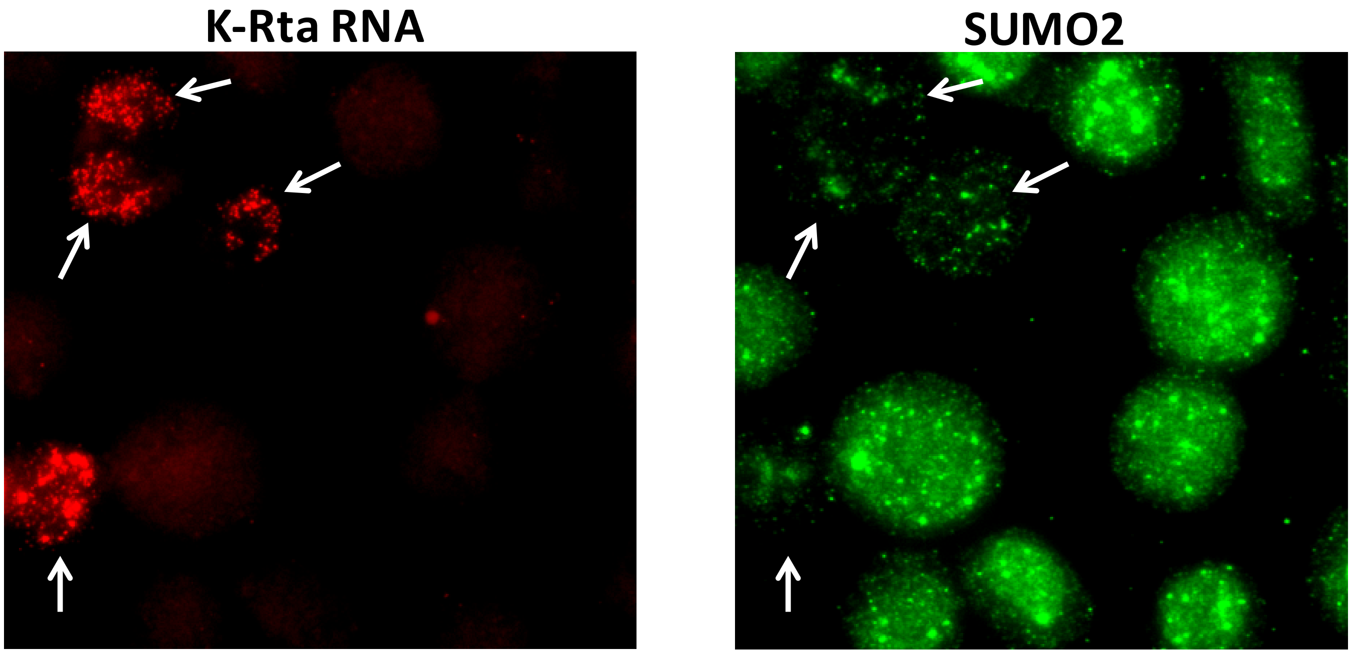

S3 Figure: Effects of RNA polymerase II translocation on SUMO. Cellular SUMO-2/3 modified proteins and/or SUMO-2/3 moiety were eliminated in KSHV reactivating cells. Immune-FISH were performed with an antibody, which recognizes both cellular SUMO-2 and SUMO-3 protein. Combination of K-Rta mediated SUMO degradation and inhibition of newly transcribing SUMO-2/3 may account for the global elimination of SUMO2/3 signals in KSHV reactivating cells.

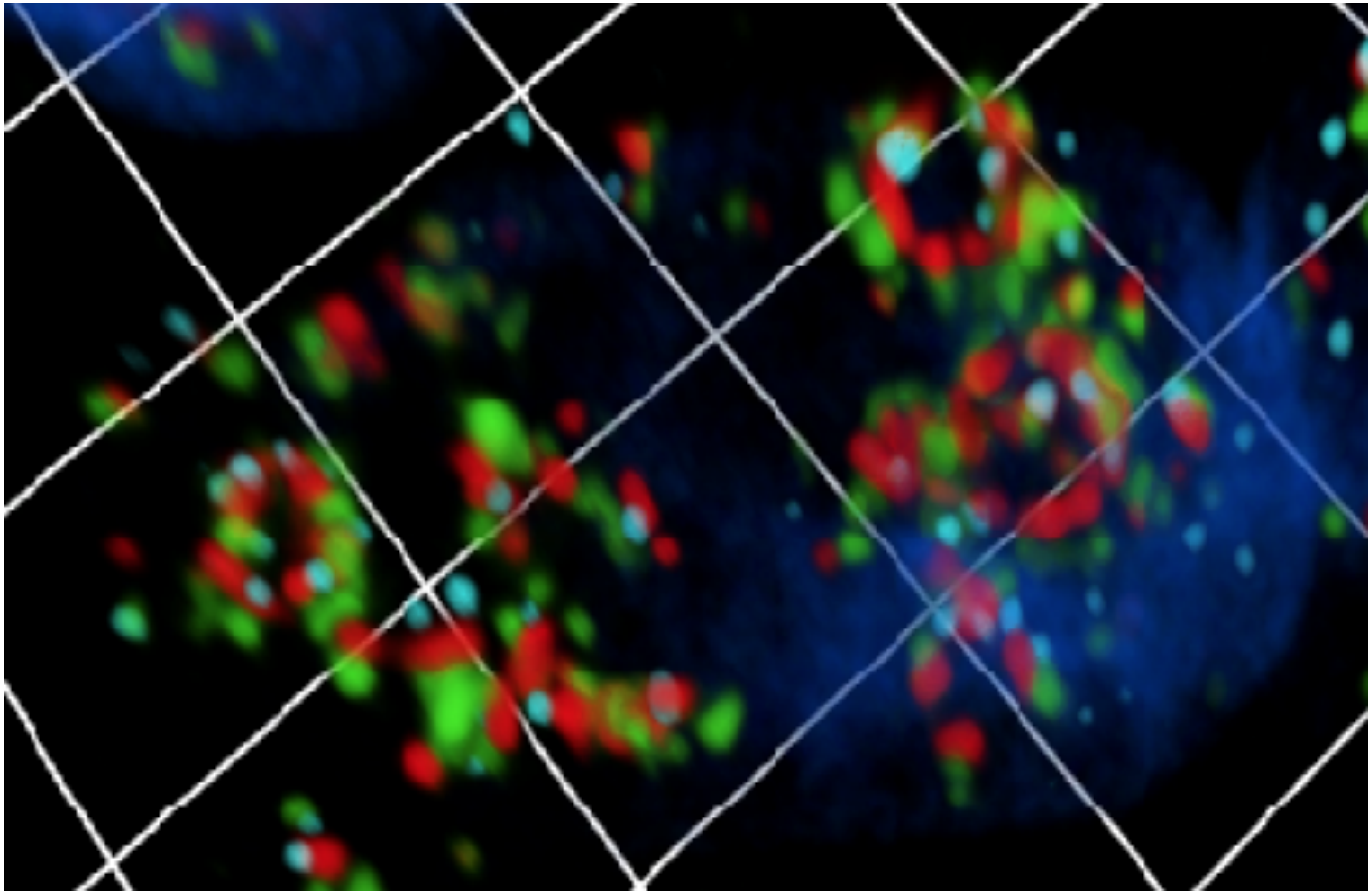

S4 Figure: Higher magnification view of KSHV transcriptional factories. Immune-FISH was performed with BCBL-1 cells. Green: LANA, Red: RNA polymerase, Light blue: K-Rta RNA.

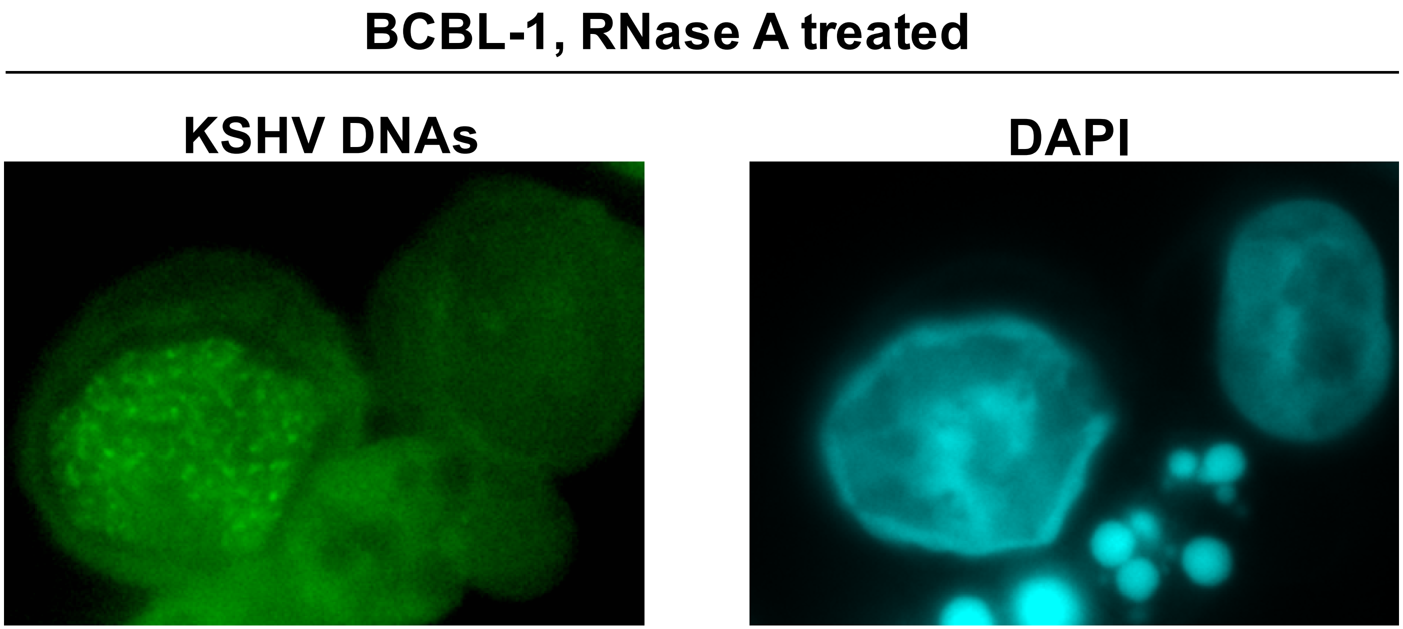

S5 Figure. DNA-FISH. BCBL-1 cells were reactivated by TPA and sodium butyrate for 48 hours. RNase A treated slide was probed with terminal repeat targeting oligos.

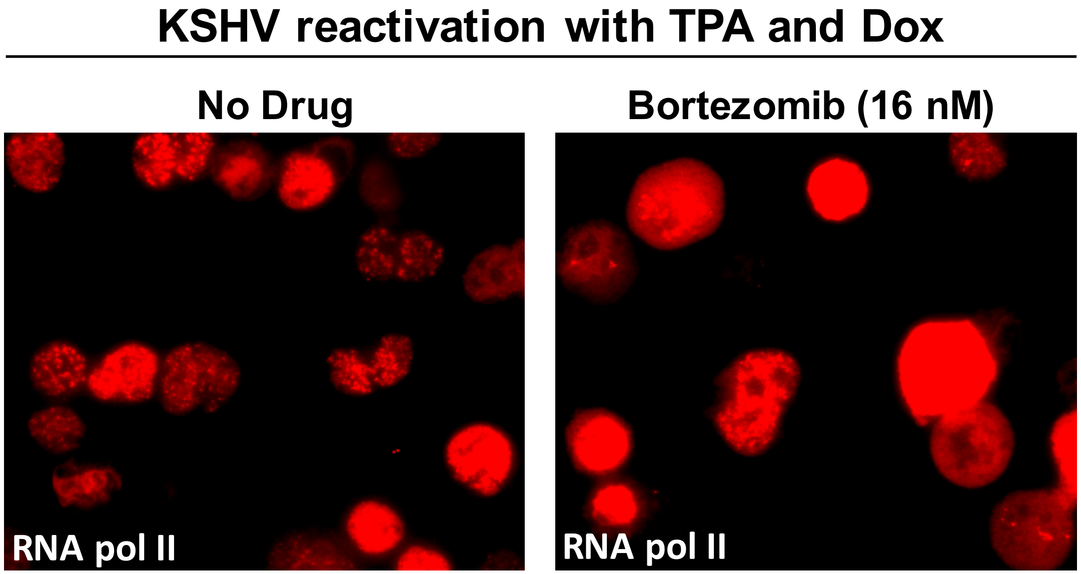

S6 Figure: Inhibition of transcriptional factory formation by proteasome inhibitor. IFA was performed with anti-RNA pol II. KSHV failed to form clear punctate RNA Pol II dots in nucleus in presence of Bortezomib (16 nM).
